# Effects of Different Litchi E-Commerce Logistics Packaging Methods on Microenvironment and Fruit Quality Variations

**DOI:** 10.3390/foods14081305

**Published:** 2025-04-09

**Authors:** Jiaming Guo, Dongfeng Liu, Guopeng Lin, Haofeng Qiu, Peng Guo, Zhiwu Ding, Dinghe Wu, Jianye Wang, Enli Lv

**Affiliations:** 1 College of Engineering, South China Agricultural University, Guangzhou 510642, China; jmguo@scau.edu.cn (J.G.);; 2 Key Laboratory of Key Technology of Southern Agricultural Machinery and Equipment, Ministry of Education, South China Agricultural University, Guangzhou 510642, China; 3 Guangzhou Liding Ecological Agriculture Development, Guangzhou 510900, China

**Keywords:** litchi preservation, foam container logistics, temperature

## Abstract

“Foam container + ice pack” is a common packaging form for e-commerce logistics of litchis. However, there are numerous factors affecting the temperature variation under this logistics mode, making it difficult to control the packaging temperature and litchi quality during the e-commerce logistics process. In order to explore the impact of the packaging scheme on the packaging environment temperature and the quality variation in litchis during the “foam container + ice pack” logistics process, this paper takes the number of ice packs, the terminal pre-cooling temperature of litchis, the weight of litchis, and whether to use aluminum foil insulating film as variable factors to study the impact rules of these factors on the EPS (Expanded Polystyrene) foam container environment temperature, the total number of fruit pericarp, and the marketable fruit rate. The experimental results show the following trends: the terminal pre-cooling temperature has a significant impact on the daily average temperature of the fruit layer; the packaging environment temperature of the 15 °C pre-cooling group on the first day and the second day is 5.00 °C and 2.78 °C higher than that of the 5 °C pre-cooling experimental group, respectively. Moreover, under this treatment, the growth rate of fruit pericarp fungi is relatively fast, which could reach 3.87 Lg (CFU/g) on the second day. Increasing the amount of litchis could maintain a lower temperature environment, but it will cause the relative conductivity increasing 4.12% compared with the groups with no weight increasing. Increasing the number of ice packs could significantly reduce the decline rate of fruit soluble solids in the first two days. The research results of this paper are expected to provide a certain reference for the quality assurance logistics and the formulation of long-distance transportation strategies for perishable agricultural products.

## 1. Introduction

Litchi belongs to the subtropical Sapindaceae family of fruits, mainly ripening in the summer, and is categorized as a non-respiratory trans-variable fruit, and its largest production area is in China, where litchi is not only abundant with nutrients, but also of high edible value [1]. Due to the strong respiration of litchi itself [2], its metabolic activity is particularly vigorous under high temperature conditions, resulting in rapid variations in the flavor and quality of the fruit [3]. A high temperature after harvesting will accelerate the enzymatic reaction as well as increase the water loss rate of litchi pericarp, which will decrease the content of anthocyanin glycosides in the pericarp, and ultimately lead to browning or even rotting [4]. As a result, the commercial value of the fruit and its edible quality will be significantly reduced. In the traditional marketing pattern, litchi typically enter fruit markets or stores in large-scale batches, at which time refrigerated trucks are often utilized to maintain their freshness [5]. However, in an e-commerce environment, consumers are mostly individuals who buy litchi mainly for personal consumption rather than for commercial purposes, so the demand is relatively small. For such small orders, using refrigerated trucks for delivery is not cost-effective. Instead, “foam container + ice packs” has become a common way of transporting litchi in e-commerce logistics [6,7].

In the meantime, this form of packaging should pay attention to some problems. Firstly, the temperature stability inside the foam container is poor, due to the accelerated melting speed of the ice packs, it is difficult to maintain a low-temperature state for a long time. In addition, there are many factors affecting the internal temperature of the foam container, making accurate prediction and control of e-commerce logistics process packaging environment temperature becomes complicated and uncertain [8]. These include, but are not limited to, the number of ice packs at the initial time, the temperature and weight of the litchi at the time of pre-cooling, the characteristics of the foam container (e.g., the material of the foam container, the degree of foaming, and the physical dimensions and thickness of the foam container, etc.), and whether or not additional insulation measures (e.g., the addition of heat-insulating film) have been used. These issues increase the possibility of transporting litchi under high temperature conditions [9]. Increasing temperature in the packaging environment will cause the vapor pressure on the surface of the litchi pericarp increase, thus prompting the water in the pericarp to migrate outward more easily, exacerbating the water loss phenomenon [10,11]. The water loss process will accelerate the degradation of anthocyanin in the litchi pericarp, leading to a decrease in the red pigment content of the litchi pericarp and browning [12]. Additionally, packing under high temperature environment enhances the activity of intracellular enzymes as well as the metabolic rate of the plant. Under such conditions, the activities of PPO (Peroxidase Oxidase) and POD (Polyphenol Oxidase) were higher [13], resulting in more vigorous physiological activities in litchi, which further accelerated the deterioration of the pericarp condition and promoted the rapid consumption of litchi’s own organic matter, ultimately affecting its flavor and the structural hardness of the fruit [14]. At the same time, the increase in temperature inside the package also accelerates the growth rate of fungus on the pericarp of litchi, triggering the problem of decay [15]. Once the litchi pericarp starts to break and rot, it will change the nutrient distribution inside the package, providing a suitable growth environment for more putrefactive fungi, leading to their massive reproduction [16]. During e-commerce logistics transportation, too much litchi will also put the lower layer under more pressure [17]. When this pressure exceeds a certain limit, it may cause friction or extrusion damage to the bottom layer of litchi, not only by increasing the risk of damage to the fruit skin, but also by providing an opportunity for the invasion of spoilage fungus [18], which ultimately results in a more serious situation of fruit rot. In addition, in the case of poor road conditions, litchi will also collide with each other to transfer vibration because of vibration, these are unfavorable to litchi e-commerce logistics preservation. According to logistics statistics, annual losses due to improper treatment in China account for about 20% of the current year’s litchi production [19,20]. In the context of pursuing the goals of “carbon peak” and subsequent “carbon neutrality”, there is a crucial need for further academic inquiry into strategies that could effectively reduce fresh fruit loss during litchi transportation and minimize cold loss associated with packaging. This necessitates a comprehensive and in-depth exploration of practical and viable solutions to enhance resource efficiency and mitigate environmental impact.

In summary, the ‘foam container + ice pack’ is a prevalent packaging method in fresh fruit e-commerce logistics, effectively extending litchi’s post-harvest shelf life. Nonetheless, this method presents unresolved issues, such as the unclear impact of packaging on environmental conditions and litchi quality making it complicated to standardized technical protocols [9]. There has been some global research on optimizing packaging and transport strategies for fruits and vegetables. Kyle et al. [21] found that foam trays reduce vibration sensitivity in apples during transit, EPS trays provided better shock protection to the apple as compared to the MF (Molded Fiber) tray, reducing the impact acceleration by more than 70%. Xie et al. [22] demonstrated that ice packs better preserve the quality of flat peaches in foam containers compared to ice films. Zhang et al. [23] advocated the use of spaced ice pack arrangements in foam containers to improve cold source utilization and maintain oyster weight. Survival of oysters transported for 2 d in this case reached 92%, Singh [24,25] and Batt [26] investigated the vibration simulation and damage response of EPS and MF foam materials on fruits and vegetables. Feng [27] examined the impact of different ice quantities on the logistics temperature of pasteurized milk during air transport, recommending a 20% ice ratio for 24 h flights and no more than 40% for durations exceeding 48 h.

Pre-cooling treatment plays a pivotal role in the “first mile” in the realization of fresh transportation of litchi [28]. Through the implementation of pre-cooling, could effectively eliminate the field heat carried by litchi and reduce its temperature, while significantly inhibiting the respiratory rate of litchi [29,30]. In view of the high specific heat capacity of litchi itself, the pre-cooled litchi has a stronger resistance to temperature increase, thus enhancing the adaptability to high-temperature environments. Therefore, the pre-cooled litchi could more effectively maintain the stability of its quality and color in the subsequent logistics distribution. Research on post-harvest pre-cooling optimization includes Zhang et al. [31], who found CWPC (Cold-water precooling) and RPC (Refrigerator precooling) methods extend yellow peach shelf life. Zhang et al. utilized CFD (Computational Fluid Dynamics) to study apple pressure differential pre-cooling efficiency, offering insights for stacking and packaging strategies. Liu et al. [32] conducted a thermodynamic analysis on cherry forced-air pre-cooling, providing recommendations for energy consumption and efficiency control. Tao et al. explored the effects of different pre-cooling methods on cabbage [33] and grape quality [34], concluding that VPC (Vacuum Pre-cooling) is suitable for cabbage preservation, while FAPC (Forced-air Pre-cooling) better delays grape softening and nutrient loss.

In conclusion, the efforts of scholars to decreasethe loss of agricultural products in the logistics process have provided valuable insights into litchi logistics and pre-cooling treatment, serving as a theoretical foundation for this study. By using the pre-cooling device [35] developed by South China Agricultural University (SCAU), experiments were designed to investigate the effects of different pre-cooling and packaging treatments on logistics temperature retention and litchi quality variation. The research aims to provide theoretical references for delaying litchi browning and decay, optimizing logistics packaging strategies, and reducing agricultural product losses.

## 2. Materials and Methods

### 2.1. Experimental Material and Design

“*Huaizhi*”, matured in early July, with dark red pericarp, juicy and sweet flesh, *Huaizhi* is one of the top four litchi varieties in China with a large market, and it has a certain application prospect to use Huaizhi as an experimental material.

The *Huaizhi litchi* used in the experiment was harvested on the same day from Conghua District of Guangzhou City and distributed at a low temperature to the College of Engineering of South China Agricultural University.

The experiment was conducted with litchi weight (L W), number of ice packs (N I C), final temperature of pre-cooling (F T P-C), and whether or not aluminum foil insulation film (AFIF) was used as variables, and the specific experimental design is shown in Table 1.

(1) Selection of fruits for experimental.

Litchi stems were trimmed, and fruits exhibiting no signs of browning or mold, with a maturity level of 80–90% and bright red appearance, were selected as experimental materials. The litchis were divided into precooling groups according to the experimental design.

(2) Pre-cooling according to experimental design.

Precooling was conducted using the pre-cooling device [35] independently developed. In order to better reflect the average pre-cooling condition, three different litchis near the center of the stack were selected to be inserted into PT100 sensors (temperature range: −200~450 °C), and the temperature variation in the core of these litchis was observed through the observation of a paperless recorder. The precooling process was terminated when the average temperature of the litchis reached the target temperature (15 °C for Group E and 5 °C for the other test groups).

(3) Sterilization of fruits by immersion.

After precooling, the litchis were immersed in a 500 μL/L dilution of prochloraz (Suzhou Formica Plant Protection Agent Co., Suzhou, China, Chemical formula: C_15_H_16_C_l3_N_3_O_2_, reagent often used for post-harvest sterilization of litchi) [36]. In order to minimize the influence of the immersion process on the pre-cooling effect, the immersion solution temperature was the same as the temperature after pre-cooling for each test group (15 °C for Group E and 5 °C for the other test groups). The immersion time for all groups was 2 min.

(4) Packaging according to experimental design

**Figure 1:**
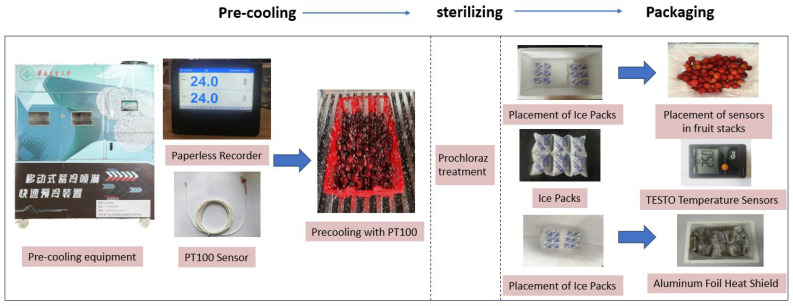
Pre-cooling and packaging process.

Following the above treatments, the litchis were packaged according to the experimental design. Ice packs were placed at the bottom of the foam containers, and the litchis, wrapped in polyethylene plastic film, were placed above the ice packs. In the experimental groups using aluminum foil insulation, both the ice packs and litchis were wrapped in 3 mm thick aluminum foil insulation before being placed in the foam containers. The ice packs, with a water storage capacity of 240 mL (6 × 40 mL), were provided by Huaying Ice Pack Factory (Wenzhou, China). The precooling and packaging processes are illustrated in Figure 1.

The experimental group with 2500 g of litchi used foam containers measuring 365 mm length × 225 mm width × 145 mm height × 22 mm thickness made of EPS. The dimensions of the foam containers used for the experimental group with 5000 g of litchi were 440 mm length × 320 mm width × 220 mm height × 22 mm thickness. After grouping, foam containers were placed at an ambient temperature of 24.8 °C. Samples were taken every two days for quality indicator measurements and the whole experiment lasted for four days.

### 2.2. Method of Experiments

#### 2.2.1. Relative Conductivity

Relative conductivity is an important indicator for the status of the membrane system within plant [37]. Plants are susceptible to rupture of cell membranes in the cases of damage, and the membrane proteins are injured, thus causing extravasation of cytoplasmic cytosol and an increase in the relative conductivity.

Three litchis were randomly selected from the experimental packaging and used a uniform hole punch on the fruit pericarp. Samples were standardized into 0.5 cm diameter disks, with a total sample weight of 2 g. The samples were immersed in distilled water for 30 min, and the initial conductivity (*D*_0_) was measured by using a DDS-307A conductivity meter (Shanghai Yidian, Shanghai, China, Range: 0.00 μS/cm to 200,000.00 μS/cm). Then, boiled the samples in water for 15 min and measured the conductivity (*D*_1_) after the sample cooling to ambient temperature. This experiment was repeated three times, as were other experiments not mentioned specifically. Relative conductivity [38] was calculated using the following formula:

(1) $$\mathrm{Relative}\;\mathrm{Conductivity}\;(\%)=\frac{D_{0}}{D_{1}}\times 100\%$$

where *D*_0_: Conductivity after 30 min of immersion, μS/cm; *D*_1_: Conductivity after 15 min of boiling, μS/cm.

#### 2.2.2. Total Soluble Solids (TSS)

Three litchis were randomly selected juiced as a group, and the juice was filtered through three layers of gauze to remove residue. A 200 μL aliquot of the juice was directly measured using a PAL-BX/ACID3 refractometer (ATAGO, Tokyo, Japan, range: 0.0~53.0%).

#### 2.2.3. Total Fungal Colony Count on Fruit Pericarp

The total fungal colony count on the litchi pericarp was determined using the plate spread count method [39]. Selected litchis were washed with 0.1% Tween 80 to obtain a microbial suspension, which was then diluted under sterile conditions. The dilutions were applied to PDA medium, sealed and incubated in a biochemical incubator at 25 °C for 3 days, followed by colony counting.

#### 2.2.4. Pericarp Color Values

The L*, a*, and b* values of the litchi pericarp were measured using a Konica Minolta CR-400 fully automatic colorimeter (KONICA MINLTA, Kyoto, Japan, range: 0.01–160.00%, pulsed xenon arc lamp (PXA)). six litchis were randomly selected for each replicate. Measurements were taken at three different locations on each litchi, and the average values were recorded. L*: Represents lightness (black to white). a*: Represents redness (green to red). b*: Represents yellowness (blue to yellow) [40].

#### 2.2.5. Browning Index and Marketable Fruit Rate

The browning level of litchis was classified according to the method described by Guo et al. [41]. Grade I and II were considered marketable, while Grade III, IV, and V fruits were considered non-marketable. Eighteen litchi were selected for browning grading in each experimental group. The criteria for rating litchi are shown in Table 2.

The browning index was calculated using Equation (2):

(2) $$\mathrm{Browing}\;\mathrm{Index}=\frac{\sum \left(Browing\;Grade*Number\;Fruits\;in\;Grade\right)}{Total\;Number\;of\;fruit}$$

The marketable fruit rate was calculated using Equation (3):

(3) $$\mathrm{Marketable}\;\mathrm{Fruit}\;\mathrm{Rate}\;(\%)=\frac{N_{0}}{N_{1}}\times 100\%$$

where *N*_0_: Total number of Grade I and II fruits. *N*_1_: Total number of fruits evaluated.

#### 2.2.6. Firmness

Fruit firmness was measured using an EPT Fruit Hardness Tester (GY-4) with the range: 0 N~30 N (Zhejiang Top Instrument, Hangzhou, China). The pericarp of the litchi fruit was plucked off and two different test positions were selected for fruit hardness testing. By increasing the pressure of the thimble until the thimble pierced the fruit, the fruit hardness was read, and the hardness of 18 litchis was measured in each group of tests, and the average value was taken.

#### 2.2.7. Average Internal Packaging Temperature

**Figure 2:**
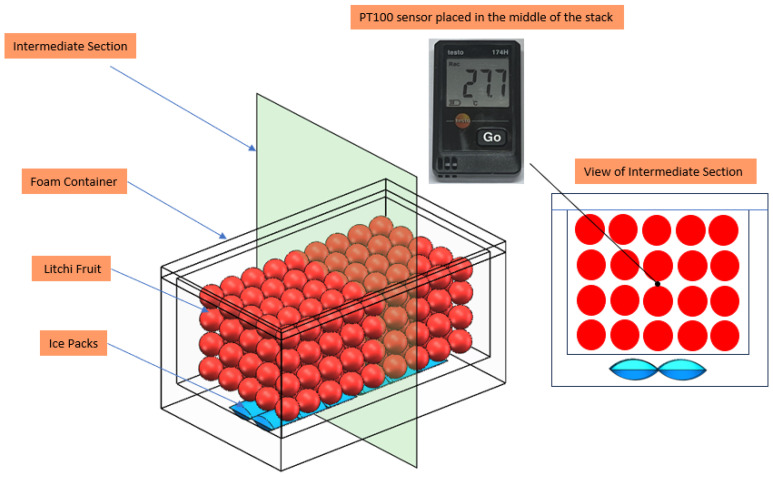
Arrangement of sensors in the package.

Testo wireless temperature sensors (TESTO AG, Titisee-Neustadt, Germany) were used to record temperature variations inside foam container, with sensors placed in the middle of the fruit layer. Temperature data were exported after the experiment, and the daily average temperature was calculated. The sensor placement is illustrated in Figure 2.

The average internal packaging temperature was calculated using Equation (4):

(4) $$\bar{T}=\frac{\sum_{i}^{n}{\bar{T}}_{i}}{n}$$

where $\bar{T}$: The average internal packaging temperature for experimental group; $\bar{T_{i}}$: Average temperature of the day recorded by the *i*th sensor; n: Number of sensors.

#### 2.2.8. Statistical Methods

The data were analyzed for significance using SPSS 26.0, selecting the Waller-Duncan multiple comparison in one-way ANOVE to analysis the significance. The statistical results were considered significant when *p* < 0.05.

### 2.3. Correlation Analysis

The correlation between different indicators is determined Pearson correlation coefficient, which is calculated as follows:

(5) $$r=\frac{\sum_{i=1}^{n}\left(x_{i}-\bar{x})(y_{i}-\bar{y}\right)}{\sqrt{{\sum_{i=1}^{n}{\left(x_{i}-\bar{x}\right)}^{2}\sum_{i=1}^{n}\left(y_{i}-\bar{y}\right)}^{2}}}$$

where *r*: Pearson’s correlation coefficient (range: −1~1, Larger absolute values indicate a stronger correlation and negative values indicate a negative correlation). $x_{i}$: The test value of indicator x; $y_{i}$: The test value of indicator y; $\bar{x}$: Average value of indicator x; $\bar{y}$: Average value of indicator y.

### 2.4. PCA Comprehensive Evaluation

The work of data dimensionality reduction and principal component division was carried out using SPSS 26.0 software. The Comprehensive Evaluation Score [42] is calculated as follows:

(6) $$F=\frac{\sum_{i=1}^{n}FACi\times \sqrt{\lambda_{i}}\times \mu_{i}}{\sum_{i=1}^{n}\mu_{i}}$$

where F: Comprehensive evaluation value of principal component analysis. FACi: Score value on component *i*. $\lambda_{i}$: Eigenvalues on the component *i*. $\mu_{i}$: Variance explanation ratios of component *i*.

## 3. Results and Analysis

### 3.1. Average Internal Packaging Temperature

The average in-package temperature variation for each test group are shown in Table 3 below.

The daily average temperature differences among the experimental groups were pronounced during the first two days. As observed in Group E, the precooling termination temperature had a substantial impact on the inside temperature. When the precooling termination temperature was 15 °C, the daily average temperature reached 16.45 °C, significantly higher than that of other groups (*p* < 0.05). Litchis possess a high specific heat capacity, meaning that lower precooling termination temperatures result in greater stored cooling capacity, which is more conducive to maintaining a low-temperature inside environment [43,44].

Group B exhibited daily average temperatures approximately 1.13 °C and 1.44 °C lower than Group A during the first two days, respectively. Increasing the weight of litchi is equivalent to enhancing the initial cold storage capacity inside the foam container, because litchi itself has a higher specific heat capacity and has been pre-cooled to have a lower temperature, which is helpful for the foam container to better maintain the low-temperature environment.

Group F averaged approximately 3.07° C and 1.87 °C less than Group A on days 0 and 1. By increasing the number of ice packs, the initial cold storage capacity of the foam container improved, which is conducive to prolonging the time of cold release and obtaining a longer-lasting low-temperature environment.

Throughout the experimental period, Groups C and D demonstrated the most effective maintenance of low-temperature conditions. On the first day, the average packaging environment temperature in Group C was 4.13 °C lower than in Group A, while Group D was 2.39 °C lower than Group B. This suggests that the use of aluminum foil insulation effectively blocks external heat from entering the logistics environment, thereby preventing temperature increases. Furthermore, the temperature difference between Groups C and D was minimal, remaining within 0.5 °C, indicating that the weight of litchis has slight impact on the logistics environment temperature when aluminum foil insulation is employed.

### 3.2. Relative Conductivity

**Figure 3:**
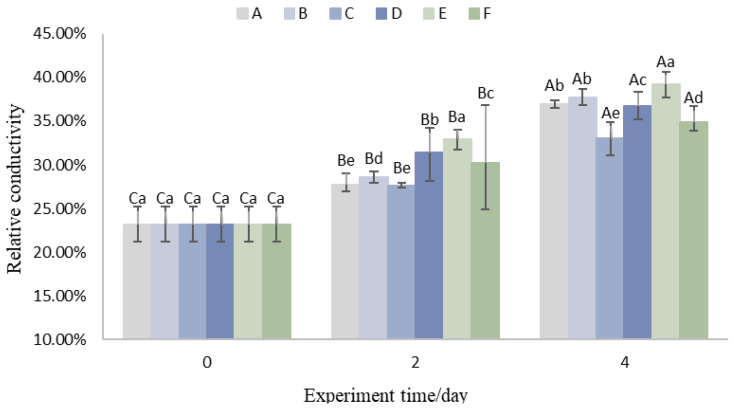
Variations in relative conductivity of the rind. (Upper case letters in the figure indicate significant differences (*p* < 0.05) demonstrated by the same treatment group at different trial times, and lower case letters indicate significant differences (*p* < 0.05) between different treatment groups at the current trial time).

Mechanical damage disrupts cell membranes, which causes an increase in membrane permeability, and electrolyte efflux from the cell improves the relative conductivity, so measuring the relative conductivity of the pericarp could also indirectly reflect the degree of pericarp damage. As shown in Figure 3, the relative conductivity of the pericarp in all experimental groups exhibited an increasing trend over storage time.

A comparison between Groups A and B, as well as Groups C and D, revealed that increasing the weight of litchis during logistics accelerated the rate of increase in relative conductivity. By the fourth day, the relative conductivity increase in Group B was 0.86% higher than in Group A, while Group D was 4.12% higher than Group C. This suggests that increasing the weight of litchis within the packaging accelerates the rise in relative conductivity, likely due to increased compression and mechanical damage to the pericarp.

Furthermore, a comparison between Groups A and C, as well as Groups B and D, indicated that Groups A and B exhibited higher relative conductivity values. This demonstrates that the use of aluminum foil insulation significantly reduces the rate of increase in relative conductivity. Group E showed the fastest rate of increase in relative conductivity among all experimental groups, reaching 39.21% by the fourth day. This suggests that lowering the pre-cooling end temperature reduced the variation in the relative conductivity of the Litchi pericarp, probably because lowering the pre-cooling temperature facilitates the maintenance of low temperatures in the packaging environment, which is an important influence on physiological activity, and enzyme activities may be inefficient because of the influence of low temperatures [13].

### 3.3. Browning Index and Marketable Fruit Rate

**Figure 4:**
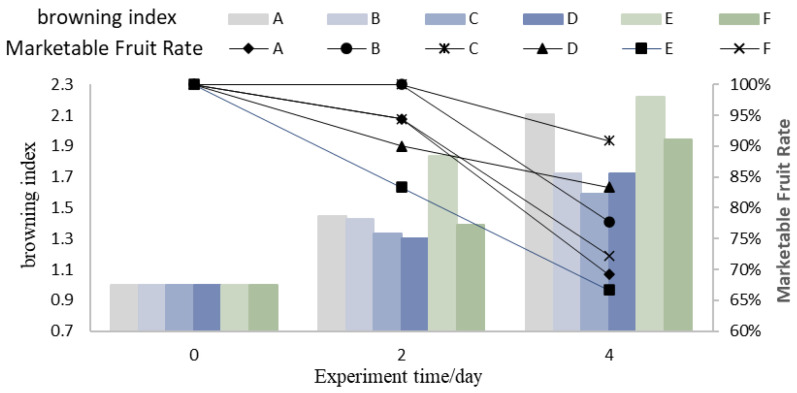
Variation in browning index and marketable fruiting rate.

**Figure 5:**
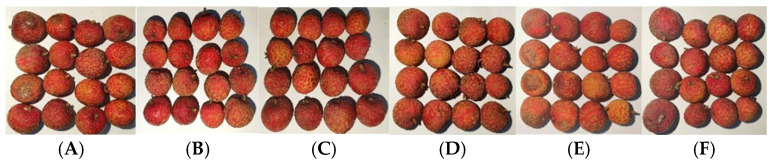
Appearance of litchi in each experimental group on day 4.

As shown in Figure 4, with the increase in storage time, the browning index of all experimental groups continuously rises, and the marketable fruit rate continuously decreases. Experimental Group E has a faster browning speed and a lower marketable fruit rate throughout the experiment. This result indicates that a higher pre-cooling temperature of the fruit is not conducive to reducing the post-harvest physiological metabolism rate of litchi, leading to an accelerated browning and fruit spoilage during the foam container logistics process, as shown in Figure 5. Additionally, compared to groups A and B, groups C and D have a slower browning speed. On the second day, the average browning index of Group C is 0.11 lower than that of Group A, and the marketable fruit rate of Group C is always higher than that of Group A. The average browning index of Group D is 0.13 lower than that of Group B, indicating that the use of aluminum foil insulation could effectively reduce the browning speed of litchi in the early stage of logistics. Comparing groups A and F, the browning index of Group F is also consistently lower than that of Group A during the 4-day storage period. On the second day, the browning degree of Group A is more severe than that of Group F, indicating that increasing the number of ice packs could also slow down the browning speed of litchi to a certain extent. The variation in the browning index of litchi basically conforms to the pattern of temperature variations within the packaging environment. When the environmental temperature increases, the physiological metabolism and water loss process in the fruit pericarp cells are accelerated [29], resulting in the decrease in anthocyanins in the cell vacuole and the acceleration of fruit browning [12].

### 3.4. Total Soluble Solids (TSS)

**Figure 6:**
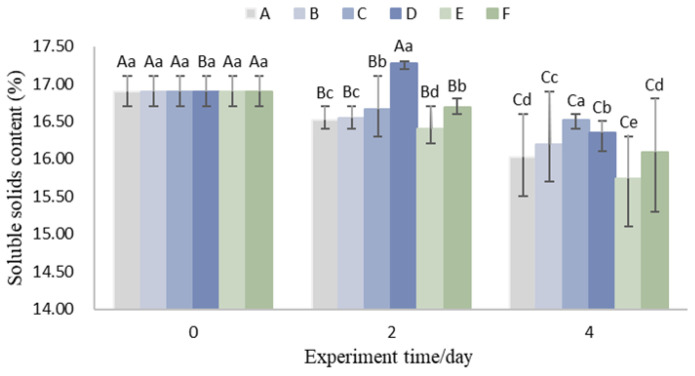
Variations in soluble solids content. (Upper case letters in the figure indicate significant differences (*p* < 0.05) demonstrated by the same treatment group at different trial times, and lower case letters indicate significant differences (*p* < 0.05) between different treatment groups at the current trial time).

As shown in Figure 6, the total soluble solids (TSS) content of litchis in all experimental groups exhibited an overall declining trend with increasing storage time. The results from Group E indicate that the TSS content in the 15 °C precooling group decreased more rapidly throughout the storage period. Compared to Group A, the TSS content in Group E was 0.23% and 0.31% lower on the second and fourth days, respectively. This is attributed to the incomplete removal of field heat, which led to heightened metabolic activity and accelerated consumption of internal substances in the litchis, consistent with the findings of Peng et al. [45].

Groups C and D, which utilized aluminum foil insulation, exhibited higher TSS content compared to Groups A and B, which did not use insulation. This suggests that maintaining a lower ambient temperature through aluminum foil insulation helps suppress litchi respiration and internal substance consumption. However, no significant patterns were observed when comparing Group A with Group B or Group C with Group D, indicating that increasing the weight of litchis has little effect on maintaining internal substance quality during logistics. As mentioned earlier, while increasing litchi weight helps maintain a low-temperature environment, the resulting compression and abrasion may enhance the permeability of plant cells, allowing more oxygen and microorganisms to accelerate internal substance degradation [46]. Thus, the impact of litchi weight on internal substance preservation during logistics is dual-sided.

In comparison to Group A, Group F, which included two additional ice packs, showed a 23% slower rate of TSS decline during the first two days. This demonstrates that increasing the number of ice packs during logistics could effectively reduce the rate of internal substance consumption in litchis.

### 3.5. Total Fungal Colony Count on Fruit Pericarp

**Figure 7:**
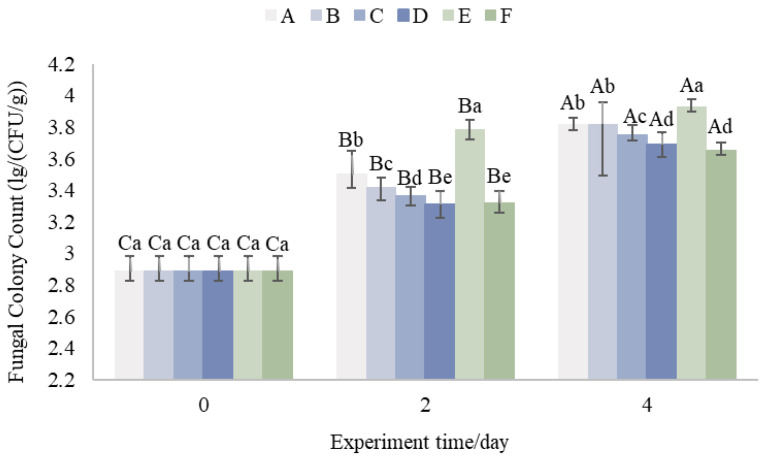
Total fungal Colony Count on Fruit Pericarp. (Upper case letters in the figure indicate significant differences (*p* < 0.05) demonstrated by the same treatment group at different trial times, and lower case letters indicate significant differences (*p* < 0.05) between different treatment groups at the current trial time).

As shown in Figure 7, the total number of fungal colonies of the pericarp increased with time. The increase in the colony number has a certain correlation with the variation in ambient air temperature in the package, and the higher average daily temperature accelerates the germination, growth and spread of fungal spores [47]. Among them, the 15 °C pre-cooling Group, Group E, reached a pericarp colony count of 3.87 Lg (CFU/g) on day 2, which was larger than the colony count of this experimental group throughout the experimental cycle, indicating that lowering the pre-cooling temperature could enhance the environmental inhibitory factors for microbial growth and thus inhibit the growth activities of microorganisms [16,48].

Group C was compared to Group A. On day 2, Group C had a lower colony count of 0.13 Lg (CFU/g) compared to Group A. Group D was compared to Group B. Group D had a lower colony count of 0.12 Lg (CFU/g) compared to Group B. The results of this study indicate that the use of aluminum foil insulating film could also enhance the environmental inhibitors of microbial growth and thus inhibit the growth activities of microorganisms. This suggests that the use of aluminum foil insulation film better reduces the risk of microbial growth during the pre-transportation period. The microbial growth rate of Group F compared with Group A decreased significantly, which indicates that increasing the number of ice packs bring about the lower temperatures and also inhibit the growth of the fungal flora.

### 3.6. Color Difference and Firmness

**Figure 8:**
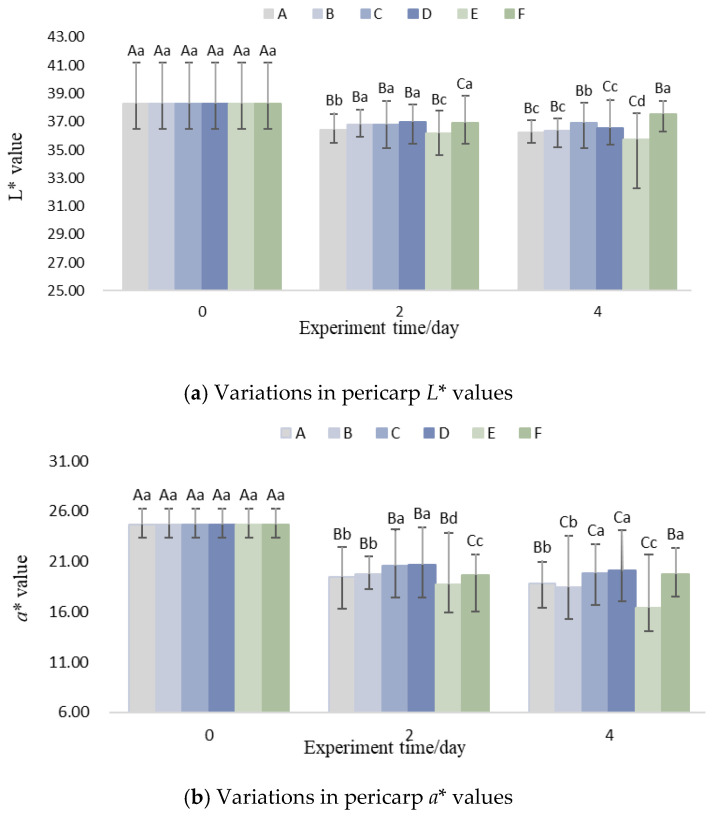

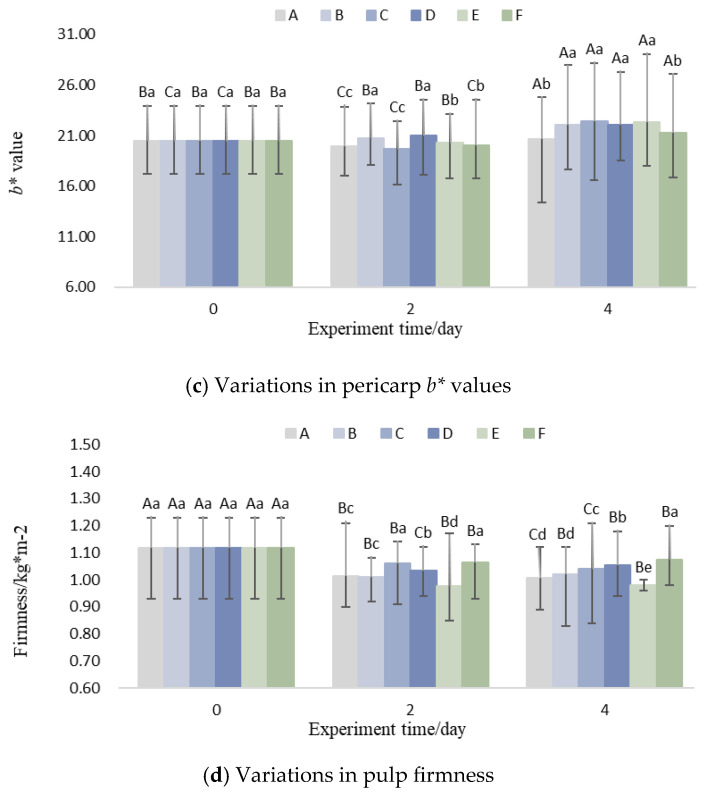
Variations in pericarp color values (L*, a*, b*) and pulp firmness. (Upper case letters in the figure indicate significant differences (*p* < 0.05) demonstrated by the same treatment group at different trial times, and lower case letters indicate significant differences (*p* < 0.05) between different treatment groups at the current trial time).

As shown in Figure 8, the lightness (L*) and redness (a*) values of litchi fruits exhibited an overall declining trend with increasing storage time, while the yellowness (b*) value showed a relatively slow increasing trend. The firmness also decreased over time.

From Figure 8a, it could be observed that the variation in lightness (L*) is correlated with the precooling termination temperature. As the precooling termination temperature decreased, the rate of decline in the lightness (L*) of litchi pericarp also accelerated. By the fourth day, the L* value of Group E decreased by 0.49° more than that of Group A.

From Figure 8b, it is evident that Groups C and D, which used aluminum foil insulation, exhibited higher a* values compared to Groups A and B, which did not use insulation. This indicates that the litchis in Groups C and D maintained better red coloration. Additionally, the precooling termination temperature significantly influenced the decline in a* values. The a* value of Group E (15 °C precooling) decreased by 8.27 over four days, while that of Group A (5 °C precooling) decreased by 5.86. The rate of decline in Group E was 41.09% faster than that in Group A, consistent with the findings of Li et al. [49].

The variation in redness (a*) is highly sensitive to temperature. Increased temperature leads to variations in vapor pressure, creating a driving force for epidermal water loss [50]. This water loss accelerates the degradation of anthocyanins in the vacuoles of litchi pericarp cells, resulting in a reduction of the a* value [12].

As shown in Figure 8c, there is a correlation between the variation in yellowness b* and the weight of litchi. A comparison between Groups A and B, as well as Groups C and D, reveals that increasing the weight of litchis accelerates the rate of increase in b* during logistics. From Figure 8d, it could be observed that the firmness gradually decreases during the logistics process. The use of aluminum foil insulation or a lower precooling termination temperature slows down the softening rate of litchis. However, increasing the weight of litchis has little effect on firmness and may instead lead to mechanical damage, resulting in color deterioration [16]. Additionally, increasing the number of ice packs could also reduce the softening rate of litchis. In summary, the variations in color difference (L*, a*, b*) and firmness of litchis are consistent with the influence of logistics strategies on temperature. Maintaining a low-temperature environment is crucial for preserving the color and firmness of litchis.

### 3.7. Correlation Analysis and PCA Comprehensive Evaluation

**Figure 9:**
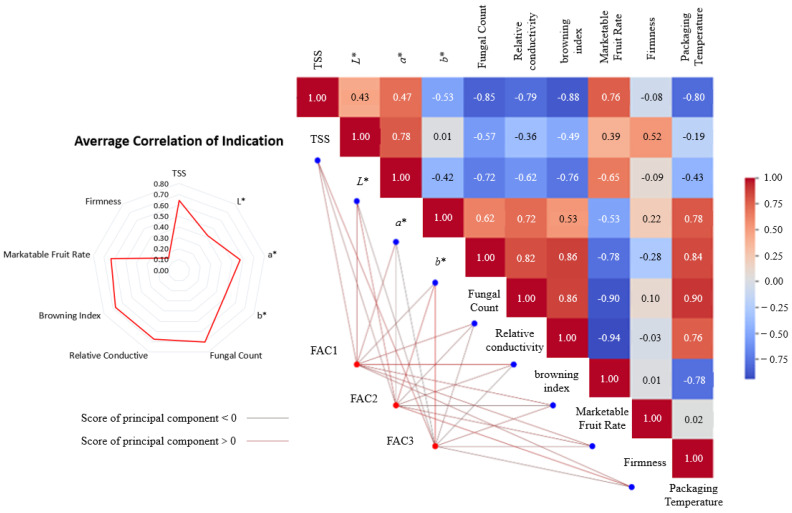
Correlation of quality indicators with principal component relationship.

As shown in Figure 9, the packaging temperature exhibited strong correlations with most quality indicators. Among these, six indicators showed correlations exceeding 0.76 with the packaging environment temperature. However, the correlations between packaging temperature and firmness (0.02) as well as pericarp lightness (L*) (0.19) were relatively weak.

Among the quality indicators, redness (a*), relative conductivity, browning index, marketable fruit rate, total soluble solids (TSS), and pericarp microbial count demonstrated significant inter-correlations, all exceeding 0.62. This indicates that there are notable interrelationships among fruit decay, browning, and pericarp relative conductivity during logistics. The variations in lightness (L*) and redness (a*) showed a correlation of 0.78, suggesting a numerical relationship between the reduction in a* and L* during the browning process. Additionally, yellowness (b*) exhibited significant correlations with pericarp microbial count and relative conductivity, implying associations among b* values, pericarp tissue damage, and microbial growth.

The pericarp microbial count, TSS content, marketable fruit rate, browning index, and relative conductivity showed average correlations greater than 0.6 with all other indicators, indicating that these metrics effectively represent overall quality variations. In contrast, firmness exhibited an average correlation of only 0.15 with other indicators, suggesting its limited association with overall quality metrics.

By the use of SPASS 26.0 software, PCA analysis was conducted on indicators such as TSS content, L*, a*, b*, epidermal microbial count, relative conductivity, browning index, good fruit rate, and firmness to establish a comprehensive evaluation index. These indicators are denoted as Q1, Q2, Q3, Q4, Q5, Q6, Q7, Q8, and Q9, respectively.

**Figure 10:**
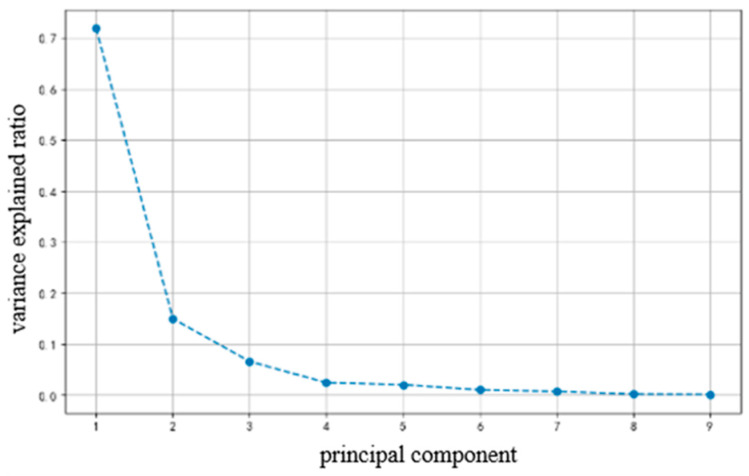
Distribution of variance explained ratio by component.

As shown in Figure 10, the quality data were standardized by the software to obtain nine feature components, of which the proportion of variance explained by component 1, component 2 and component 3 were 71.95%, 14.97 and 6.59%, respectively, for a total of 93.51%. This indicates that these three components could respond well to the characteristics of litchi quality, and the use of these three components for the comprehensive evaluation of different experimental treatments could have a favorable representation. The eigenvalues of FAC1, FAC2, and FAC3 are 8.42, 1.75, and 0.77, respectively.

Table 4 presents the loadings of each quality indicator on FAC1, FAC2, and FAC3.

Table 5 shows the final score of each experimental group. The value of F is the score obtained by weighing the variance explanation ratio and eigenvalue of the principal component. The subscript in the experimental group column represents the corresponding experimental time in days. From the data in the comprehensive score table, it could be seen that on both the 2nd and 4th day, the comprehensive quality score of Group E is lower. This indicates that ensuring the pre-cooling temperature is an important link in the foam container logistics process, and the residual field heat will significantly accelerate the deterioration speed of litchi quality. On the 2nd day of logistics, groups C and D, which used aluminum foil insulation film, performed better. When the logistics time came to the 4th day, Group F with 4 ice packs performed better than groups C and D. This may be because the aluminum foil insulation layer reduces the heat transfer rate between the litchi logistics environment and the outside world. When the logistics time is long and the internal low temperature could no longer be maintained, the respiratory heat generation of litchi itself cannot be dissipated to the outside, but will more easily cause the temperature rise in the packaging environment in the later stage. This phenomenon could also be observed in Table 3. In addition, at the early stage of logistics, the weight of litchi has little effect on the variation in comprehensive quality, but in the later stage of logistics, it will accelerate the decline speed of comprehensive quality.

## 4. Discussion

In the multifaceted and dynamic realm of e-commerce logistics, effectively preserving the quality of litchis has emerged as an urgent and pivotal issue to address. The research findings of this paper suggest that strategies such as reducing the final pre-cooling temperature, increasing the number of ice packs, enhancing litchi weight, and utilizing aluminum foil insulating films are considered to have positive effects on environmental and quality maintenance. However, the actual efficacy of these measures is far from being simply intuitive, necessitating an in-depth exploration of their underlying mechanisms and potential impacts.

Reducing the final precooling temperature could eliminate the field heat carried by litchis. Rapid cooling could effectively slow down the metabolic rate of litchis, thereby delaying the process of quality decline [29,30]. According to the research results in this paper, the browning index of litchis after 15 °C precooling treatment is 0.39 higher than that of 5 °C treatment on the second day. However, it should be noted that excessively low temperatures may cause irreversible damage to the cellular structure of litchis, leading to the leakage of intracellular substances, which in turn affects the taste and nutritional value of litchis [51]. The research results of Hu et al. [52] found that precooling temperatures below 5 °C could cause chilling injury to the pericarp of litchis, resulting in increased permeability of the pericarp membrane and relative conductivity. Therefore, when determining the final precooling temperature, it is necessary to carefully balance the relationship between the cooling rate and the risk of cellular damage.

Increasing the number of ice packs and the weight of litchi could enhance the low temperature maintenance function and provide a more stable cold storage environment for litchi. Ice packs work by absorbing surrounding heat and slowly releasing cold, which helps to maintain a stable temperature during transportation. In the results of this paper, the average package temperature on the day of storage in the four ice pack test group was 3.07 °C lower than that in the two ice pack group. Increasing the weight of litchi could also reduce the packaging temperature, it is difficult to achieve the cooling effect of the ice packs, on the day of storage, the average packaging temperature of the 5000 g Group B was 1.13 °C lower than that of the 2500 g Group A. Although increasing the amount of lichi could increase the initial cold storage capacity in the package, lichi and ice packs are different, lichi couldn’t release cold through the phase change, the ice in the ice packs in the liquefaction of the phase change will show latent heat properties, and could absorb more heat. In addition, the experimental results of this paper show that increasing the weight of lichi accelerates the change in indicators such as pericarp b* value and hardness value, which is obviously not good. By increasing the weight of lichi, the lichi inside the package has to face more crushing problems, especially those stacked in the lower layer, which will increase the risk of mechanical damage to the pericarp and microbial growth during storage. In addition, the results of the study by Fadiji et al. [7] concluded that the degree of mechanical damage increases when agricultural products are transported in the presence of vibration, and the effects of the increase in the weight of the lichi are greater, which accelerates the changes in the appearance and quality of the lichi, which is exposed to a greater risk of mold and browning.

The use of aluminum foil insulation could reduce the growth of fungi inside the package. The experimental results showed that at 2 days of storage, the number of fungi in the experimental group using aluminum foil insulation film was less than that of the unused group. The use of aluminum foil insulation film could better prevent the exchange of heat between the inside and outside of the package in order to maintain the low temperature inside the package. The results of the study by Si et al. [53] showed that temperature is the most important factor affecting the growth of microorganisms in agricultural products, high temperature will accelerate the reproduction and metabolism of microorganisms, and the appearance of the agricultural products in the process of continuous change will also increase the microorganisms of the environmental capacity, and ultimately lead to the decay of the agricultural products. Therefore, in the actual logistics process, the use of aluminum foil heat insulation film to increase the heat insulation capacity of the foam box is of great significance to the preservation of lichi logistics.

Subsequent research should deeply analyze the specific relationship between mechanical damage of litchi and transportation parameters, including transportation speed, road surface factors, litchi weight and packaging structure, etc. It is necessary to carry out research on vibration mitigation strategies for the logistics process in response to litchi’s unique resonance frequency.

## 5. Conclusions

In this paper, the effects of litchi weight, pre-cooling temperature, the use of aluminum foil insulation film and the number of ice packs on the physiological and packaging environmental changes in litchi were discussed by experimental methods. The main conclusions are as follows:

(1) Increasing the weight of pre-cooled litchi, increasing the initial number of ice packs, reducing the final pre-cooling temperature and using aluminum foil insulation film could better maintain the low temperature in the foam container environment. Among them, the pre-cooling temperature has a greater impact on the ambient temperature. The average packaging environment temperature of the 15 °C pre-cooling end temperature group is 5.00 °C higher than that of the 5 °C pre-cooling treatment group.

(2) The variation in final pre-cooling temperature has significant effects on browning index, marketable fruit rate, soluble solid content, a* value, L* value and epidermal microbial count. Compared with the variations in logistics factors in other experimental groups, the variation in final pre-cooling temperature has the greatest impact on these indicators.

(3) More litchi causes an increase in the relative conductivity of the pericarp. Compared with the 5000 g group and the 2500 g group, the maximum absolute difference in relative conductivity in four days of storage could reach 4.12%. In addition, increasing the weight of litchi will increase the yellowness b* value and cause variations in fruit color.

(4) Increasing the number of ice packs could alleviate the browning speed of litchi pericarp. When the number of ice packs increases from 2 to 4, the consumption speed of soluble solids in litchi decreases by 23%. In addition, increasing the number of ice packs also has a certain inhibitory effect on the growth of epidermal microbial count in the first two days of logistics.

(5) The average temperature in the packaging environment on the day when the aluminum foil insulation layer is used is less than 7.9 °C. Under the condition of 5 °C pre-cooling, the use of aluminum foil insulation layer could further reduce the heat variation between the inside and the outside. In addition, this measure could also reduce the epidermal microbial count by about 0.12 Lg (CFU/g) on the second day due to its early low-temperature effect. In the later stage of storage, it could delay the decline of redness a* value and reduce the speed of browning and fruit decay.

## Figures and Tables

**Table 1:** Different packaging and pre-cooling treatment.

Group	F T P-C/°C	N I C/Pouch	L W/g	AFIF
A	5	2	2500	Unused
B	5	2	5000	Unused
C	5	2	2500	Used
D	5	2	5000	Used
E	15	2	2500	Unused
F	5	4	5000	Unused

**Table 2:** Litchi Fruit Browning Grade Criteria.

Grade	Criteria
Grade I	Bright red pericarp or sporadic brown spots at the tips of the scales.
Grade II	Browning area less than one-third of the pericarp surface; moderate appearance.
Grade III	Browning area between one-third and one-half of the pericarp surface; poor appearance.
Grade IV	Browning area greater than one-half of the pericarp surface; partial red color; poor appearance.
Grade V	Entire pericarp brown or juice leakage; no red color or dark red appearance.

**Table 3:** Variation in daily average temperature in package (°C).

Group	Day 0	Day 1	Day 2	Day 3	Day 4
A	11.45 ± 0.24	20.16 ± 0.09	25.66 ± 0.03	25.63 ± 0.15	25.72 ± 0.14
B	10.32 ± 1.21	18.72 ± 0.21	26.15 ± 0.02	26.49 ± 0.51	26.83 ± 0.36
C	7.62 ± 0.21	17.06 ± 0.06	25.16 ± 0.24	25.64 ± 0.25	26.85 ± 0.13
D	7.93 ± 0.31	16.84 ± 0.50	24.65 ± 0.44	26.64 ± 0.55	26.79 ± 0.36
E	16.45 ± 0.65	22.94 ± 0.92	25.91 ± 0.25	26.34 ± 0.56	25.28 ± 0.46
F	8.38 ± 2.01	18.29 ± 0.93	25.12 ± 0.02	25.28 ± 0.19	25.29 ± 0.28

**Table 4:** Principal component loadings.

	Q_1_	Q_2_	Q_3_	Q_4_	Q_5_	Q_6_	Q_7_	Q_8_	Q_9_
FAC1	0.3299	0.3231	0.3622	−0.2204	−0.3667	−0.3624	−0.3741	0.3371	0.2960
FAC2	−0.2129	0.4406	0.2608	0.5037	−0.1159	0.2609	0.1224	−0.3102	0.4961
FAC3	0.442	−0.1246	−0.0456	0.7331	0.1555	0.1011	−0.3048	0.3049	−0.1711

**Table 5:** Composite scores for experimental groups.

Group	FAC1	FAC2	FAC3	F1	F2	F3	F	Rankings
A_2_	18.62	28.43	16.77	53.99	37.53	14.75	45.44	5
B_2_	18.68	28.08	17.30	54.18	37.06	15.23	45.53	4
C_2_	19.33	28.76	16.50	56.05	37.97	14.52	46.96	1
D_2_	19.29	27.63	17.74	55.94	36.47	15.61	46.74	2
E_2_	17.76	28.09	16.93	51.51	37.08	14.91	43.60	6
F_2_	1893	27.99	16.79	54.90	36.95	14.78	46.00	3
A_4_	17.48	27.99	16.89	50.70	36.95	14.86	42.99	4
B_4_	17.31	28.63	18.12	50.18	37.78	15.95	42.81	5
C_4_	18.13	29.31	18.46	52.59	38.69	16.24	44.70	2
D_4_	18.10	29.14	18.10	52.49	38.47	15.93	44.57	3
E_4_	15.90	28.01	18.09	46.12	36.98	15.92	39.77	6
F_4_	18.26	29.14	17.20	52.96	38.47	15.14	44.87	1

## Data Availability

The original contributions presented in the study are included in the article, further inquiries can be directed to the corresponding author.
